# Characterizing Virulence of the *Pyrenophora tritici-repentis* Isolates Lacking Both *ToxA* and *ToxB* Genes

**DOI:** 10.3390/pathogens7030074

**Published:** 2018-09-12

**Authors:** Jingwei Guo, Gongjun Shi, Zhaohui Liu

**Affiliations:** Department of Plant Pathology, North Dakota State University, Fargo, ND 58108, USA; jingwei.guo@ndsu.edu (J.G.); gongjun.shi@ndsu.edu (G.S.)

**Keywords:** wheat leaf spot, host-selective toxins, pathogenicity test, QTL mapping

## Abstract

The fungus *Pyrenophora tritici-repentis* (*Ptr*) causes tan spot of wheat crops, which is an important disease worldwide. Based on the production of the three known necrotrophic effectors (NEs), the fungal isolates are classified into eight races with race 4 producing no known NEs. From a laboratory cross between 86–124 (race 2 carrying the *ToxA* gene for the production of Ptr ToxA) and DW5 (race 5 carrying the *ToxB* gene for the production of Ptr ToxB), we have obtained some *Ptr* isolates lacking both the *ToxA* and *ToxB* genes, which, by definition, should be classified as race 4. In this work, we characterized virulence of two of these isolates called B16 and B17 by inoculating them onto various common wheat (*Triticum aestivum* L.) and durum (*T*. *turgidum* L.) genotypes. It was found that the two isolates still caused disease on some genotypes of both common and durum wheat. Disease evaluations were also conducted in recombinant inbred line populations derived from two hard red winter wheat cultivars: Harry and Wesley. QTL mapping in this population revealed that three genomic regions were significantly associated with disease, which are different from the three known NE sensitivity loci. This result further indicates the existence of other NE-host sensitivity gene interactions in the wheat tan spot disease system.

## 1. Introduction

Tan spot is a devastating foliar disease on both common wheat (*Triticum aestivum* L.) and durum wheat (*T*. *turgidum* L.) [1]. This disease is caused by the fungal pathogen *Pyrenophora tritici-repentis* (*Ptr*), which belongs to the family of dothideomycetes in ascomycete. The typical symptoms incited by this pathogen on susceptible cultivars include tan-colored, elliptical-shaped necrotic lesions, which are often surrounded by chlorotic halos [2]. Under favorable conditions, the lesions can coalesce, which forms a large area of dead leaf tissue. These symptoms can be indicative of the necrotrophic nature of lifestyle where the fungus may produce necrotrophic effectors to cause the death of plant cells.

Since the 1980s, several studies have revealed that the symptoms of necrosis and chlorosis induced in the host by *Ptr* were genetically distinct [3,4,5]. These studies also led to the development of a wheat differential set for tan spot, a lesion type disease rating scale, and a basic race classification system, which have been widely adopted today. Later, the fungal pathogen was known to produce host selective toxins now called necrotrophic effectors (NEs) to induce necrosis or chlorosis symptoms by interacting with their corresponding host sensitivity genes. Up to date, three fungal-produced NEs have been identified, which are Ptr ToxA (NCBI accession ID: AAB61464.1), Ptr ToxB (NCBI accession ID: AAO73337.1), and Ptr ToxC, which interact with the wheat genes *Tsn1*, *Tsc2*, and *Tsc1*, respectively [6,7]. Therefore, eight races have been designated according to their ability to produce single or a combination of the three necrotrophic effectors (NEs). Races 2, 3, and 5 produce one NE, Ptr ToxA, Ptr ToxC, and Ptr ToxB, respectively, which causes disease on differential lines Glenlea (necrosis), 6B365 (chlorosis), and 6B662 (chlorosis), respectively. Races 1, 6, and 7 produce a combination of two NEs with Ptr ToxA and Ptr ToxC for race 1, Ptr ToxB and Ptr ToxC for race 6, and Ptr ToxA and Ptr ToxB for race 7. Race 8 produces all three NEs while race 4 produces no known NEs [1,8]. However, the current race classification in *Ptr* has been challenged by the identification of isolates that do not conform to any of the eight races [9,10]. It has been reported that *Ptr* likely produces additional NEs in addition to the three previously described [1,11,12].

*Ptr* is a homothallic fungus, which means it can be sexually reproduced by self-crossing. This precludes the possibility to further identify fungal virulence factors through genetic analysis in *Ptr*. Recently, we have developed a new method for the fungal cross and genetic mapping in *Ptr* [13]. The race 2 isolate 86–124 and race 5 isolate DW5 were only known to produce Ptr ToxA (encoded by the *ToxA* gene, ID: 5983599) and Ptr ToxB (encoded by the *ToxB* gene ID:AY243460), respectively, but they have been shown to produce other unknown NEs [1,7]. We have obtained some isolates from the cross between 86–124 and DW5 that do not produce any of the three NEs. These isolates should be classified as race 4 according to the current classification system. However, our hypothesis is that these isolates produce other unknown NEs and are still virulent. In this work, we test the hypothesis by using the pathogenicity test on various common and durum wheat genotypes and further map the host factors that interact with these unknown NEs or virulence factors, which are present in these isolates.

## 2. Results

### 2.1. Pathogenicity Test

**Figure 1:**
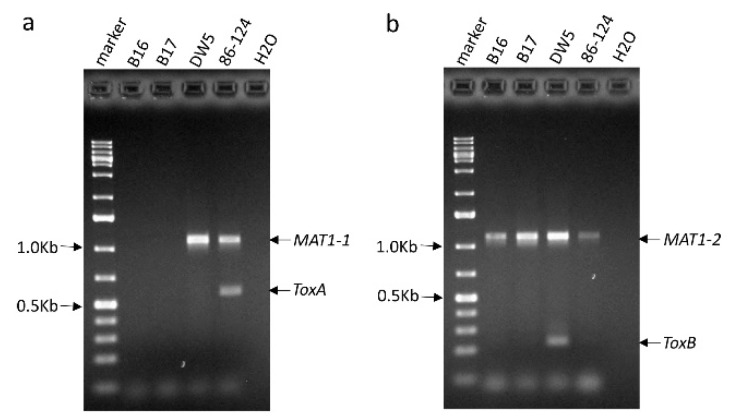
PCR testing of the presence of the *ToxA* and *ToxB* genes in *Pyrenophora tritici-repentis* isolates B16 and B17. Parental isolates 86–124 and DW5, which were used to obtain B16 and B17 as well as water control (H_2_O) were also included in the PCRs (**a**) *ToxA* was multiplexed with the *MAT1-1* gene. (**b**) *ToxB* was multiplexed with the *MAT1-2* gene.

In our previous study, we used genetically modified mating type isolates 86–124∆MAT1-2 (only carry *MAT1-1*) and DW5∆MAT1-1 (only carry *MAT1-2*) to develop a segregating population and obtained several isolates that had neither *ToxA* nor *ToxB* gene. From these isolates, we randomly selected two of them: B16 and B17 for this study. Using gene specific primers (see methods), we confirmed that the two isolates lacked both *ToxA* and *ToxB* genes (Figure 1a,b). As expected, the *ToxA* and *ToxB* genes were amplified from 86–124 and DW5, respectively. Given the two isolates derived from the cross of 86–124∆*MAT1-1* and DW5∆*MAT1-2*, they should harbor only one mating type gene. The result showed both of them have *MAT1-2*, but not *MAT1-1* (Figure 1a,b).

**Figure 2:**
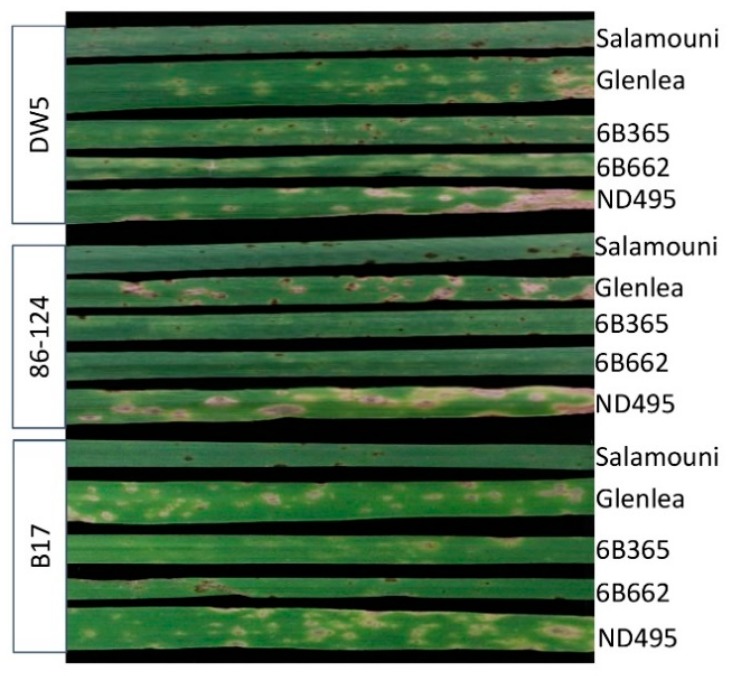
Disease reaction of wheat tan spot differential lines to fungal inoculation with B17, DW5, and 86–124. Tan spot differentials included Salamouni (universal resistant), Glenlea (sensitive to Ptr ToxA), 6B365 (sensitive to Ptr ToxC), and 6B662 (sensitive to Ptr ToxB). The hard spring wheat line ND495 was included as a susceptible control. The secondary leaves of the lines were photographed 7 days after inoculations. B16 has similar reactions as B17 toward differential lines. Therefore, the photo was not shown.

B16 and B17 were first tested on tan spot differential lines including Salamouni, Glenlea, 6B365, and 6B662 as well as the highly susceptible line ND495. To compare the difference in virulence, 86–124 and DW5 were also inoculated onto these lines side-by-side with B16 and B17. As shown in Figure 2, 86–124 and DW5 caused high levels of disease on ND495 and their differential lines Glenlea (necrotic lesions) and 6B662 (chlorotic lesions), respectively. Compared to 86–124 and DW5, B16 and B17 were similar in their virulence, which causes fewer diseases due to the development of smaller sizes of necrotic lesions on Glenlea and ND495 (Figure 2). This is likely due to the fact they do not produce Ptr ToxA and Ptr ToxB.

B16 and B17 were further tested on a set of wheat lines including common wheat (*Triticum aestivum* L.) and durum wheat genotypes (*T*. *turgidum* L.) (Table 1). The majority of these lines are hard red spring wheat and durum wheat cultivars in the Northern Great Plains of the United States. Disease mean from three replications for each line was listed in Table 1 and the least significant difference (LSD) were calculated among those lines.

Significant difference was observed in disease means between some lines, according to the LSD values. The most common wheat lines had a disease mean equal or lower than 2.00, which indicates a resistance reaction. A few common wheat lines such as Mott, Steele-ND, Harry and Barlow had a disease mean greater than 4.00, which is considered to be highly susceptible. All the durum wheat lines had disease means greater than 3.00, which indicates that they are moderately or highly susceptible to a tan spot caused by B16 and B17 (Table 1).

### 2.2. QTL Mapping

**Figure 3:**
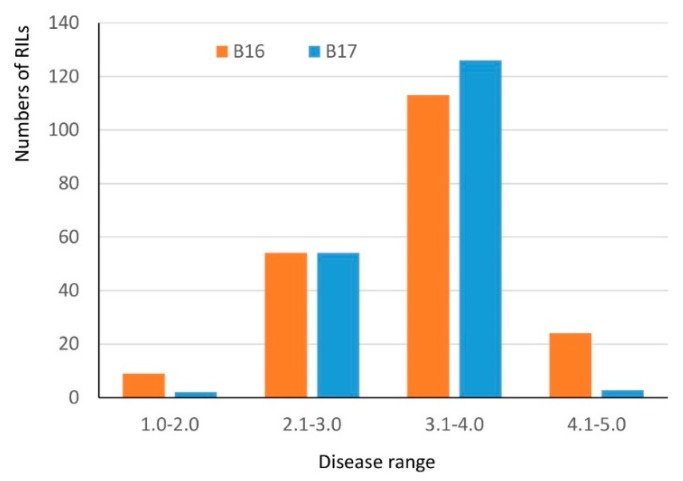
Histogram of disease reaction distribution of the HW population inoculated with B16 and B17. The *x*-axis indicates the specific ranges in the 1–5 scale and the *y*-axis indicates the number of lines within each range.

**Figure 4:**
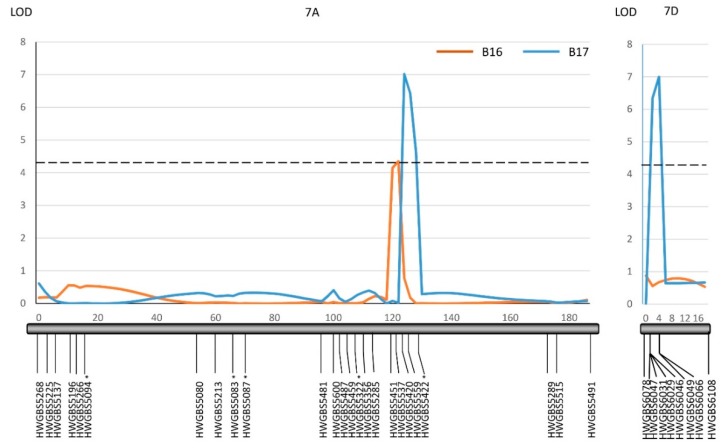
Composite interval mapping of QTL on chromosomes 7A and 7D associated with a reaction to B-16 and B-17 in the HW population. QTL lines were drawn for B16 (orange) and B17 (blue). The closed black bars represent individual linkage groups with marker loci shown on the bottom and a genetic position in cM is shown on the top. A *dash line* represents the logarithm of the odds (LOD) significance threshold of 4.2. The LOD and *R*^2^ value for each QTL are presented in Table 2. The asterisk indicates markers that showed a segregation distortion.

Both Harry and Wesley are hard red winter cultivars from Nebraska. A recombinant inbred line population (the HW population) has been developed from the cross between Harry and Wesley. A high-quality genetic linkage map has been constructed in this population using SNP markers generated from genotyping-by-sequencing. The constructed genetic linkage maps contained 3,641 SNP markers covering all 21 wheat chromosomes and spanning 1959 cM in the genetic distance with the marker density at 1.8 cM per marker. The maps have been successfully applied to locate QTL associated with important agronomic traits (see methods). Harry and Wesley differed greatly in the reaction to B16 and B17 with Wesley being resistant and Harry being highly susceptible (Table 1). Therefore, the HW population was evaluated for reacting to tan spots caused by the two isolates individually to identify host factor(s) interacting with the virulence factors in B16 and B17. The results showed the population segregated from highly resistant to high susceptible in the reaction to the two isolates (Figure 3). Shapiro-Wilk tests indicated an acceptance of a normal distribution hypothesis for a disease reaction to B17 (*p* = 0.1011), but a rejection of a normal distribution hypothesis for B16 (*p* = 0.0012). The disease means of the population were 3.4 and 3.3 for B16 and B17, respectively. Composite interval QTL mapping revealed three genomic regions designated as *QTs.zhl.-7A-1*, *QTs.zhl.-7A-2*, and *QTs.zhl.-7D*, which are significantly associated with reactions to tan spots caused by these two isolates and the resistance alleles for these QTL all come from Wesley (Table 2, Figure 4). *QTs.zhl.-7A-1* was the only QTL identified for B16 inoculation and it explained 8% of disease variations caused by this isolate. *QTs.zhl.-7A-2* and *QTs.zhl.-7D* were identified for B17 inoculation with a similar LOD value (7.0) and explained 9% and 11% of disease variations, respectively. *QTs.zhl.-7A-1* and *QTs.zhl.-7A-2* are very close for the positions in the map and they could be the same QTL (Figure 4).

## 3. Discussion

The tan spot disease is well known to involve three fungal-produced NE-host sensitivity gene interactions including Ptr ToxA-*Tsn1*, Ptr ToxB-*Tsc2*, and Ptr ToxC-*Tsc1* [7]. Many studies have shown that all three interactions can play significant roles in the development of tan spots in diverse wheat germplasm and populations [1,14,15,16]. However, several lines of evidence have suggested that the disease system is not only based on these three NE-sensitivity gene interactions. Friesen et al. (2003) developed a mutant at the *Tsn1* gene using ethyl methanesulfonate (EMS) treatment and found that even though the *tsn1* mutant is insensitive to Ptr ToxA, it was still highly susceptible to 86–124, which is only known to produce Ptr ToxA [17]. Even though some isolates of races 3 and 5 are known to produce only chlorosis—inducing NE, they were found to induce necrosis on specific common and durum wheat genotypes [18,19,20]. In a study to compare the difference in virulence between a race 1 isolate and its *ToxA* mutant, See et al. (2018) did not observe any significant disease reduction on some Australian wheat varieties [21]. Many QTL mapping studies have identified genomic regions other than three known sensitivity loci (*Tsn1*, *Tsc1*, and *Tsc2*) [1,14,16]. All these strongly suggested that the disease system involves additional NE-sensitivity gene interactions or other types of interactions. In this study, we used two fungal isolates that produce no known NEs and showed that they still caused disease on some durum and common wheat lines. We also identified host genetic factors that interact with the unknown NEs in these two isolates using host QTL mapping. Our work further demonstrates that the fungal pathogen produces additional NEs or other types of virulence factors besides the three known ones.

Our work also demonstrates that the current race classification system is not sufficient. In the current system, race 4 does not produce any known NEs, which means it is avirulent to all the wheat lines in the differential set. Both B16 and B17 do not produce any of the known NEs. Therefore, by definition, it should be classified as race 4. However, our pathogenicity tests showed they still caused disease on Glenlea as well as ND495 even though they had a lower level of virulence compared to 86–124 and DW5. A few studies have also identified new isolates that cannot be classified using the current race classification system [10,22,23,24]. To solve the problem, the new NEs or virulence factors and their corresponding host genetic factors need to be identified. The tan spot differential set should be expanded to include the lines harboring the host genetic factor(s) that interact with the new NE(s) or virulence factor(s). Using QTL mapping, we identified three wheat genomic regions that are associated with a reaction to the two isolates. These genomic regions must harbor the host genes that could directly or indirectly interact with the new NE(s) or virulence factor(s) in B16 and B17. After these, QTL are confirmed in diverse genetic backgrounds, the RILs from the HW population, which harbor the individual QTL, could be used in a differential set to detect the new NE.

We identified one QTL (*QTs.zhl.-7A-*1) for B16 inoculation and two QTL (*QTs.zhl.-7A-2* and *QTs.zhl.-7D*) for B17 inoculation with *QTs.zhl.-7A-*1 and *QTs.zhl.-7A-*2 possibly being the same. The slight shifting of the QTL peaks may be due to the mis-phenotyping in disease reading. The results suggested that B16 and B17 may carry different unknown NEs or virulence factors, but they may also harbor common unknown NE. We observed the recombination and segregation of the *ToxA* and *ToxB* genes in the progeny derived from the cross between 86–124 and DW5 [13]. Likewise, the other unidentified NEs or virulence factors would also segregate in the population if they were polymorphic between 86–124 and DW5. Although B16 and B17 lack both *ToxA* and *ToxB* genes, they could differ in the presence of other NEs, which leads to the identification of different set QTL in the HW population for the two isolates. A total of eighteen isolates were obtained from the cross, which do not have neither the *ToxA* nor *ToxB* gene. It would be interesting to test all these isolates onto the HW population or other segregating populations. By doing that, we can potentially catalog all the new NEs that are segregating in the progeny from the 86–124 × DW5 cross.

In addition to the NE sensitivity loci *Tsn1* on 5BL and *Tsc2* on 2BS, QTL associated with tan spot disease have been mapped to other wheat chromosomes or regions for both 86–124 and DW5 using bi-parental or association mapping [14,15,16,25,26,27,28]. It is possible that the QTL we identified on 7D (*QTs.zhl.-7D*) for B17 may be the same as the one identified by Gurung et al. [25] for DW5. If so, the NE interacting with this QTL might come from DW5, not 86–124. However, there is no tan spot resistance or susceptible QTL reported on the chromosome 7A for either 86–124 or DW5. Therefore, it remains unknown whether the NE interacting with *QTs.zhl.-7A-1* or *QTs.zhl.-7A-2* come from 86–124 or DW5. Epitasis of the Ptr ToxA-*Tsn1* interaction over other interactions has been reported in the wheat-*Ptr* pathosystem [21,29]. Thus, it is possible that the effect of the new NE that interacts with *QTs.zhl.-7A-1* or *QTs.zhl.-7A-2* could be masked by the Ptr ToxA-*Tsn1* interaction and the 7A QTL has not been previously detected for 86–124. Therefore, the progeny lacking both *ToxA* and *ToxB* genes would be useful to identify new NEs because there is no interference from the three known NE-sensitivity gene interactions.

## 4. Materials and Methods

### 4.1. Fungal Isolates and PCR Testings

The Ptr isolate B16 and B17 were derived from a fungal cross between 86–124 (race 2) and DW5 (race 5) that were modified for the mating type system [13]. We obtained a total of 18 progeny that have neither *ToxA* nor *ToxB* gene and randomly selected two of them, which includes B16 and B17 for the characterization of fungal virulence outside of the three known NEs. To confirm the presence of the *ToxA* and *ToxB* genes, the gene specific primers that have been published previously were used (Table 3). Since the two isolates were derived from the mating type gene modified strains of 86–124 (race 2) and DW5 (race 5), they were also tested for the presence of the mating type genes using the corresponding primers (Table 3). The fungal isolates were grown on V8-PDA for 7 days and then the mycelium was collected for DNA extraction by gently scratching the surface of the cultures. Genomic DNA extraction was done after following the processes described by Shjerve et al. [30]. In PCR testing, the *ToxA* primers were multiplexed with those for the *Mat1-1* gene and primers for the *ToxB* gene were multiplexed with those for *Mat1-2*. The multiplex PCRs were performed according to the protocols by Ameen et al. [13]. 86–124 and DW5 were also used as controls in PCR and the pathogenicity tests.

### 4.2. Plant Materials, Experimental Designs, and Disease Evaluations

A total of 32 wheat lines including common wheat and durum wheat genotypes as well as the tan spot differential lines (Salamouni, Glenlea, 6B365, and 6B662) and susceptible check ND495 were used in pathogenicity tests. To map host genetic factors interacting with the new virulence factor, we used a bi-parental wheat population derived from the cross between the two Nebraska hard red winter wheat cultivars Harry and Wesley. The population, referred to as the HW population, consisted of 178 recombinant inbred line (RILs) and was kindly provided by Dr. Stephen Baenziger at the University of Nebraska, Lincoln, NE, USA. All the inoculation experiments were carried out in the greenhouse room with a temperature ranging from 23 to 28 °C and a growth chamber with a setting at 21 °C and a 12-h photoperiod. All the wheat lines and the RILs were planted in small cones with three seeds per cone (Stuewe and Sons, Inc., Corvallis, OR, USA) filled with Sunshine SB100 soil (Sun Grro Horticulture, Bellevue, WA, USA) supplied with Osmocote Plus 15-19-12 fertilizer (Scotts Sierra Horticultural Product Company, Maysville, OH, USA). All the cones were arranged in RL98 trays (Stuewe and Sons, Inc., Corvallis, OR, USA). The winter wheat cultivar Jerry was planted as the border to reduce the edge effect [28]. The plants were grown in the greenhouse room and were inoculated at the two to three leaf stages, which were approximately two weeks from the planting. Fungal inoculum preparation, fungal inoculation, and post inoculation incubation of plants followed the method described in Lamari and Bernier [3]. For each line, six seeds were planted in two cones with six plants in each experiment. The inoculation experiment was repeated three times and, within each experiment, a randomized complete block design (RCBD) was used to arrange the plants. Disease ratings were conducted using the 1–5 scale with 1 being highly resistant and 5 being highly susceptible, which was described by Lamari and Bernier [3].

### 4.3. Statistic Analysis and QTL Mapping

In the pathogenicity tests, the NPAR1WAY procedure was performed to compute exact p-values for the simple linear rank statistics based on Wilcoxon scores with the disease readings that were collected from different replications and experiments. The ordinary disease data was transformed into parametric data using PROC RANK followed by ANOVA and t tests of the least significant difference (LSD) in SAS 9.4 Software (SAS Institute, 2016) to detect the significance of the difference among different wheat genotypes. Normal distribution of the disease data for each isolate was evaluated with the Shapiro-Wilk test using t PROC UNIVARIATE in SAS 9.4 (SAS Institute, 2016). Disease means for each RIL from three replications were used for QTL mapping. The genetic linkage maps of the HW population have been published containing 3,641 SNP markers and covering all 21 wheat chromosomes and has been successfully applied to the QTL mapping of flag leaf related traits [33]. The same map was used in this study to locate QTL for tan spot resistance. The QTL mapping was performed using PC-based software QGene 4.0 [34]. The composite interval mapping function installed in the software was used for QTL analysis. A permutation test with 1000 iterations yielded an LOD threshold of 4.2 at a *p* = 0.05 experiment-wise significance level.

## 5. Conclusions

Our work further demonstrates that the *Ptr*-wheat pathosystem is not just based on the three necrotrophic effector-host sensitivity interactions, but likely involves additional NE-sensitivity interactions or other types of host-pathogen interactions.

## Figures and Tables

**Table 1:** Reaction of wheat genotypes to the tan spot caused by the isolates B16 and B17.

Genotypes	Wheat Type	B16 ^a^		B17 ^a^	
		Disease Mean	Rank Mean	Disease Mean	Rank Mean
Rollag	Common wheat	1.17	6.17O	1.17	9.83JK
Velva	Common wheat	1.17	6.17O	1.50	17.50JK
Jenna	Common wheat	1.50	11.50NO	1.50	17.50JK
RB07	Common wheat	1.50	14.50MNO	1.50	17.50JK
Brennan	Common wheat	1.67	17.17MNO	1.67	21.33IJK
Glenn	Common wheat	1.83	25.33KLMN	1.50	18.33IJK
Briggs	Common wheat	2.00	28.50JKLM	2.33	38.17GH
Chinese Spring	Common wheat	2.00	28.50JKLM	2.50	23.00FG
Prosper	Common wheat	2.00	28.50JKLM	1.33	13.67JK
Vantage	Common wheat	2.00	28.50JKLM	2.17	33.67GHI
Wesley	Common wheat	2.00	28.50JKLM	2.00	29.33HIJ
Howard	Common wheat	2.17	33.67IJKL	2.17	33.67GHI
Kelby	Common wheat	2.50	42.83HIJ	2.50	43.00FG
Select	Common wheat	2.50	42.83HIJ	2.50	43.00FG
SY Soren	Common wheat	2.67	46.83GHI	2.67	47.50EFG
SY Tyra	Common wheat	3.00	56.00FGH	3.50	67.50CD
Mott	Common wheat	4.00	81.50BCD	4.00	80.00BC
Steele-ND	Common wheat	4.00	81.50BCD	4.00	80.00BC
Harry	Common wheat	4.00	81.50BCD	4.50	96.00A
Barlow	Common wheat	4.1	86.67ABC	4.33	90.67AB
Alkabo	Durum wheat	2.17	33.67IJK	2.67	47.50EFG
Lebsock	Durum wheat	3.00	56.00FGH	3.50	66.00CD
Grenora	Durum wheat	3.00	56.00FGH	3.17	59.67DE
Carpio	Durum wheat	3.1	60.00EFG	3.17	59.67DE
Tioga	Durum wheat	3.5	68.50DEF	3.50	66.00CD
Ben	Durum wheat	3.8	77.00BCD	4.00	80.00BC
Dilse	Durum wheat	4.00	81.50BCD	4.00	80.00BC
Divide	Durum wheat	4.00	81.50BCD	4.00	80.00BC
Langdon	Durum wheat	4.33	91.83AB	4.50	96.00A
Rusty	Durum wheat	4.33	91.83AB	4.33	90.67AB
Mountrail	Durum wheat	4.50	97.00A	4.50	96.00A
Pierce	Durum wheat	4.50	97.00A	4.50	96.00A
Least Significant Difference			15.0		15.70

^a^ Disease was rated using a 1–5 scale with 1 being highly resistant and 5 being highly susceptible. Data represented mean values of 12 individuals. Because disease scores are ordinary data, disease means were transformed into rank means using PROC RANK in the SAS program for mean separation. In the rank mean column, means with the same letter are not significantly different.

**Table 2:** QTL associated with a reaction to the tan spot caused by B16 and B17 in a recombinant inbred line population derived from Harry and Wesley.

QTL	Interval (cM)	Flanking Markers	B16 ^a^		B17 ^a^		Source ^b^
			LOD	*R* ^2^	LOD	*R* ^2^	
*QTs.zhl.-7A-1*	120.0-122.0	*HWGBS5451-HWGBS5420*	4.4	0.08	-	-	W
*QTs.zhl.-7A-2*	124.0-130.0	*HWGBS5420-HWGBS5422*	-	-	7.0	0.11	W
*QTs.zhl.-7D*	176.0-180.0	*HWGBS6047-HWGBS6066*	-	-	7.0	0.09	W

^a^ Permutation test with 1000 iterations yielded a LOD value of 4.2 and it was used as the cut-off to identify significant QTL. *R*^2^ values indicate the amount of the phenotypic variation explained by the QTL. ^b^ Source indicates which parental line contributes the resistance allele with H being Harry and W being Wesley.

**Table 3:** List of primers used in this study.

Primers	Sequences (from 5′ to 3′)	Purpose	Reference
PtrPLP7	GCTTTACTACAACTTTCCTCTACC	Amplify the *MAT1-2*	Lepoint et al. (2010) [31]
PtrPLP10	GTACGGGCCAGCATGACGTGC		
PtrPLP2	CAGAACAAAGGCAGGACTGTGAGC	Amplify the *MAT1-1*	Lepoint et al. (2010) [31]
PtrPLP4	ATGCGCTCAGCAAGGAAGGTCG		
TA51F	GCGTTCTATCCTCGTACTTC	Amplify the *ToxA*	Andrie et al. (2007) [32]
TA52R	GCATTCTCCAATTTTCACG		
TB71F	GCTACTTGCTGTGGCTATC	Amplify the *ToxB*	Andrie et al. (2007) [32]
TB6R	ACGTCCTCCACTTTGCACACTCTC		
